# Prenatal Organochlorine and Methylmercury Exposure and Memory and Learning in School-Age Children in Communities Near the New Bedford Harbor Superfund Site, Massachusetts

**DOI:** 10.1289/ehp.1307804

**Published:** 2014-08-06

**Authors:** Sara T.C. Orenstein, Sally W. Thurston, David C. Bellinger, Joel D. Schwartz, Chitra J. Amarasiriwardena, Larisa M. Altshul, Susan A. Korrick

**Affiliations:** 1 Department of Epidemiology, Harvard School of Public Health, Boston, Massachusetts, USA; 2 Channing Division of Network Medicine, Department of Medicine, Brigham and Women’s Hospital, Harvard Medical School, Boston, Massachusetts, USA; 3 Department of Biostatistics and Computational Biology, School of Medicine and Dentistry, University of Rochester, Rochester, New York, USA; 4 Children’s Hospital, Harvard Medical School, Boston, Massachusetts, USA; 5 Department of Environmental Health, Harvard School of Public Health, Boston, Massachusetts, USA; 6 Environmental Health and Engineering, Inc., Needham, Massachusetts, USA

## Abstract

Background: Polychlorinated biphenyls (PCBs), organochlorine pesticides, and methylmercury (MeHg) are environmentally persistent with adverse effects on neurodevelopment. However, especially among populations with commonly experienced low levels of exposure, research on neurodevelopmental effects of these toxicants has produced conflicting results.

Objectives: We assessed the association of low-level prenatal exposure to these contaminants with memory and learning.

Methods: We studied 393 children, born between 1993 and 1998 to mothers residing near a PCB-contaminated harbor in New Bedford, Massachusetts. Cord serum PCB, DDE (dichlorodiphenyldichloroethylene), and maternal peripartum hair mercury (Hg) levels were measured to estimate prenatal exposure. Memory and learning were assessed at 8 years of age (range, 7–11 years) using the Wide Range Assessment of Memory and Learning (WRAML), age-standardized to a mean ± SD of 100 ± 15. Associations with each WRAML index—Visual Memory, Verbal Memory, and Learning—were examined with multivariable linear regression, controlling for potential confounders.

Results: Although cord serum PCB levels were low (sum of four PCBs: mean, 0.3 ng/g serum; range, 0.01–4.4), hair Hg levels were typical of the U.S. fish-eating population (mean, 0.6 μg/g; range, 0.3–5.1). In multivariable models, each microgram per gram increase in hair Hg was associated with, on average, decrements of –2.8 on Visual Memory (95% CI: –5.0, –0.6, *p* = 0.01), –2.2 on Learning (95% CI: –4.6, 0.2, *p* = 0.08), and –1.7 on Verbal Memory (95% CI: –3.9, 0.6, *p* = 0.14). There were no significant adverse associations of PCBs or DDE with WRAML indices.

Conclusions: These results support an adverse relationship between low-level prenatal MeHg exposure and childhood memory and learning, particularly visual memory.

Citation: Orenstein ST, Thurston SW, Bellinger DC, Schwartz JD, Amarasiriwardena CJ, Altshul LM, Korrick SA. 2014. Prenatal organochlorine and methylmercury exposure and memory and learning in school-age children in communities near the New Bedford Harbor Superfund Site, Massachusetts. Environ Health Perspect 122:1253–1259; http://dx.doi.org/10.1289/ehp.1307804

## Introduction

Polychlorinated biphenyls (PCBs) were formerly used as nonflammable dielectrics in electronic parts, vehicles for pesticide application, pigment suspension agents in carbonless copy paper, lubricants, and plasticizers in caulk and sealants (Korrick and Altshul 1998). Dicholorodiphenyldichloroethylene (DDE) is the major degradation product of dichlorodiphenyltrichloroethane (DDT), a pesticide that was widely used in the United States and is still in use elsewhere for malaria control. PCBs, DDE, and other organochlorines are lipophilic and therefore accumulate in fatty tissue, biomagnifying up the food chain. They readily cross the placenta, placing the developing fetus at risk for exposure.

Like organochlorines, methylmercury (MeHg) bioaccumulates and crosses the placenta. The usual source of MeHg is consumption of contaminated fish and marine mammals, particularly larger long-lived predators at the top of the food chain. There have been several mass human poisoning incidents resulting from high levels of prenatal MeHg or PCB exposures in which psychomotor and cognitive delays were observed in the children, while the mothers remained relatively unaffected (Amin-Zaki et al. 1974; Harada 1978; Lai et al. 2001). Thus, the developing nervous system of the fetus appears to be especially vulnerable to the effects of these neurotoxicants.

There is evidence supporting particular susceptibility of memory and learning abilities to early life PCB and MeHg exposure (Darvill et al. 2000; Jacobson and Jacobson 1996; Oken et al. 2008). Furthermore, memory and learning skills are important to school performance and later life productivity (Sheslow and Adams 1990). Existing epidemiologic literature, however, is inconclusive with respect to the effect of modest prenatal PCB exposure on childhood memory and learning among general population samples (Gladen and Rogan 1991; Jacobson and Jacobson 1996; Jacobson et al. 1990, 1992; Longnecker et al. 2003; Vreugdenhil et al. 2004). Findings from studies of the effects of prenatal MeHg on childhood memory and learning, across a range of exposures, are also inconsistent (Davidson et al. 2006; Grandjean et al. 1997; Oken et al. 2008; Palumbo et al. 2000). Although there is less literature investigating the effect of DDE exposure on memory and learning, results are inconclusive (Eskenazi et al. 2006; Gladen and Rogan 1991; Torres-Sánchez et al. 2013). There is evidence of effect modification between prenatal exposure to PCBs and MeHg in determining measures of childhood neurodevelopment, but this interaction has not been consistently observed (Grandjean et al. 2001; Stewart et al. 2003). Last, many previous studies of organochlorines and MeHg were done in populations with relatively high levels of exposure due to dietary habits such as frequent consumption of contaminated fish (Davidson et al. 2001; Jacobson and Jacobson 1996; Stewart et al. 2003) or marine mammals (Ethier et al. 2012; Grandjean et al. 2001), making the generalizability of results to more commonly experienced lower exposures uncertain.

Although PCBs have been banned in the United States since the late 1970s, reservoirs exist in building structures and in soil, often in locales frequented by children, such as public schools (Herrick et al. 2004). Additionally, though dietary fish consumption is the primary exposure route for MeHg and is an important source of PCBs, fish contains many nutrients that are beneficial for pregnant women and developing fetuses. These potential nutritional confounders have not been consistently considered in prior literature.

Therefore, our study considers the association of joint low-level prenatal organochlorine and MeHg exposures, adjusted for potential nutritional confounders, with school-age assessments of memory and learning.

## Methods

*Study population*. The New Bedford Harbor was highly contaminated with PCBs from industrial waste disposal by local electronics manufacturing beginning in the 1940s through 1977 (Choi et al. 2006). The New Bedford Cohort study was designed to assess the relation of prenatal PCB, pesticide, and metal exposure with child development. A sample of 788 mother–infant pairs were recruited from St. Luke’s Hospital in New Bedford, Massachusetts, at the time of the infant’s birth, between 1993 and 1998. There are approximately 2,000 births at the hospital per year, about 10% of which met the study eligibility criteria and occurred when study examiners were available for recruitment and examination of newborns. All mothers were ≥ 18 years of age and resided in one of four towns surrounding the contaminated harbor—New Bedford, Acushnet, Fairhaven, or Dartmouth. Families were primarily English speaking, though Portuguese study materials were available for the six Portuguese speakers. Mothers who had a cesarean section birth or delivered infants requiring high-grade neonatal care or who were not available for neonatal examination were excluded. Study children were assessed at birth, later infancy, and 8 years. This analysis focuses on the 607 children who were assessed at 8 years.

*Organochlorine exposure assessment*. Prenatal organochlorine exposure was estimated by measuring levels of 51 PCB congeners and *p,p*´-DDE in cord serum collected at birth. Analyses were performed at the Harvard School of Public Health Organic Chemistry Laboratory using high-resolution gas chromatography with a capillary column and electron capture detection (Korrick et al. 2000).

The limit of detection (LOD) for individual PCBs ranged from 0.001 to 0.04 ng/g serum, with most < 0.01 ng/g (Korrick et al. 2000). For DDE, the LOD was 0.07 ng/g serum (Korrick et al. 2000). Quantifiable values below the LOD were used to optimize statistical power and avoid biased exposure estimates associated with censoring at the detection limit (Kim et al. 1995). Reproducibility was high, with a 3% within-batch coefficient of variation and a 20% between-batch coefficient of variation for the sum of PCBs and similar performance for DDE.

Two PCB measures were used in our analysis: *a*) the sum of the four most prevalent PCBs (ΣPCB_4_) (congeners 153, 118, 138, 180), which were measured with the least measurement error and are most frequently used to investigate congener-specific effects; and *b*) the weighted sum (toxic equivalent or TEQ) (Van den Berg et al. 2006) of the measured dioxin-like PCBs (mono-*ortho* congeners 105, 118, 156, 167, and 189) to assess congeners that may have a different toxicological action from non-dioxin-like PCBs. Because of insufficient serum volume for sample-specific lipid measures, concentrations of the ΣPCB_4_ were measured on a wet-weight basis. We used the average serum lipid content of discarded cord blood from the study hospital to lipid adjust dioxin-like PCBs so their measurement scale would be comparable to other studies (Sagiv et al. 2010).

*MeHg exposure assessment*. Prenatal MeHg exposure was assessed by measuring the total mercury (Hg) concentration in maternal peripartum hair. Hair Hg is a valid biomarker of MeHg exposure because MeHg enters hair follicles in quantities directly proportional to levels in the blood, is incorporated into the hair shaft (World Health Organization 1990), and does not appear to change in concentration once incorporated (Myers and Davidson 1998). Hair was collected approximately 10 days after delivery, and the proximal 3 cm was analyzed to estimate exposure in the last trimester. The last trimester may be the most important exposure window with regard to many aspects of neurodevelopment (Grandjean et al. 1999a; Kershaw et al. 1980). When the proximal (cut) end of the hair could not be determined (45% of samples), a random 3-cm segment was analyzed. Of note, average hair Hg levels of those with and without a known cut end were comparable (mean, 0.63 μg/g; range, 0.03–5.13 for those with a known cut end; mean, 0.64 μg/g; range, 0.07–3.04 μg/g for those without a known cut end). Although the Hg concentration of hair may be affected by external contamination, hair treatment, and growth rate (Suzuki 1988), hair treatment is less likely to affect hair close to the scalp (Ohba et al. 2008). Samples were washed before analysis to remove any external contamination. Hair Hg analysis was performed using direct mercury analysis methods for atomic absorption at the Harvard School of Public Health Trace Metals Laboratory (Oken et al. 2005). Laboratory recovery rates for quality control standards were 90–110%, precision was > 95%, and the average LOD was 0.05 μg/g of hair. None of the hair Hg measures were below the LOD.

*Memory and learning assessment*. At 8 years of age, 607 (78% of eligible) study children participated in neuropsychological testing at our study clinic. Testing included the Wide Range Assessment of Memory and Learning (WRAML), which assesses immediate and delayed recall of verbal and visual material, and yields an overall general memory score and domain-specific indices of visual memory, verbal memory, and learning (Sheslow and Adams 1990). The WRAML consists of nine subtests, which are age-scaled, summed, and standardized using manual norms to create the Verbal Memory Index, the Visual Memory Index, and the Learning Index, each with a mean ± SD of 100 ± 15.

*Covariate assessment*. After birth, a trained study nurse reviewed hospital records to obtain infant race/ethnicity, birth weight, and gestational age (based on mother’s last menstrual period), as well as information regarding the mother’s pregnancy and delivery, and the baby’s initial pediatric evaluation. An obstetrical (OB) risk score was derived to summarize any adverse conditions before or during pregnancy, birth, and the neonatal period (Hobel et al. 1973; Sokol et al. 1977).

At a 2-week home visit, mothers were interviewed to obtain information on maternal diet, including consumption of fish before and during pregnancy using a food frequency questionnaire (Salvini et al. 1989); smoking, alcohol, and drug use; medical and reproductive histories; infant feeding; and detailed demographic (race/ethnicity, income) and occupational and educational histories for both parents. An estimate of prenatal omega-3 intake was calculated using self-reported intake from the food frequency questionnaire and the average omega-3 content of reported foods.

The study assessment at 8 years of age included a home visit to assess the quality of the home environment and parenting skills using the Home Observation for Measurement of the Environment (HOME) (Caldwell and Bradley 1985). Maternal IQ was measured using the Kaufman Brief Intelligence Test (KBIT) (Kaufman and Kaufman 1990), and maternal depression symptoms were assessed using the Beck Depression Inventory (BDI-II) (Beck et al. 1996). A questionnaire interview of the mothers or primary caregivers was conducted to update information obtained at infancy. Blood lead levels measured as part of routine pediatric screenings between 9 months and 4 years of age were abstracted from medical records.

*Statistical analysis*. We used bivariate analysis to examine the relationship of the four primary exposure measures (ΣPCB_4_, dioxin-like PCBs, DDE, and MeHg) with the three primary outcome measures (Verbal Memory Index, Visual Memory Index, and Learning Index). Residual plots were examined to assess assumptions of homoscedasticity and normality of the residuals, and Cook’s distances were used to assess whether any observations were unusually influential. Results of these regression diagnostics confirmed the appropriateness of using multivariable linear regression models with untransformed variables for our analyses.

Potential covariates considered in our models included examiner (two examiners were used); child age at examination, sex, race/ethnicity, birth year, birth weight, gestational age, school grade, peak blood lead level from ages 1 to 3 years; parental education at birth; household income at birth; OB risk score; HOME score at 8 years of age; and maternal parity, age at birth, race/ethnicity, birth place, marital status at birth, breastfeeding, prenatal smoking, prenatal alcohol, drug use in the year before birth, prenatal omega-3 consumption, prenatal fish consumption, IQ, and depression (modeled as continuous or categorical variables, as shown in Table 1).

**Table 1:** Descriptive characteristics of included versus excluded New Bedford Cohort children born 1993–1998 and assessed with the WRAML at 8 years of age.

Descriptive characteristic	Included^*a*^			Excluded^*b*^		
	*n* (%)	Mean ± SD	Range	*n* (%)	Mean ± SD	Range
Outcome measures
Verbal Memory Index	393	88.3 ± 13.2	48–126	209	87.4 ± 14.6	52–130
Visual Memory Index	393	91.2 ± 13.0	52–133	209	91.4 ± 12.4	57–119
Learning Index	393	97.5 ± 14.0	55–135	209	97.8 ± 13.8	60–142
Exposure measures
∑PCB_4_ (ng/g serum)^*c*^	393	0.3 ± 0.3	0.01–4.4	192	0.3 ± 0.3	0–2.6
Dioxin-like PCBs (pg TEQ/g lipid)^*d*^	393	1.5 ± 2.9	0–42.8	192	1.4 ± 1.7	0.1–12.0
DDE (ng/g serum)	393	0.5 ± 1.1	0.02–14.9	192	0.5 ± 0.9	0–10.2
Hair Hg (μg/g)	393	0.6 ± 0.6	0.03–5.1	27	0.5 ± 0.3	0.1–1.2
Child characteristics
Age at examination (years)	393	8.1 ± 0.6	7.1–11.0	209	8.2 ± 0.7	7.2–10.7
Birth weight (g)*	393	3,384 ± 431	2,242–5,221	209	3,454 ± 451	1,901–4,767
Gestational age (weeks)	391	39.7 ± 1.3	33.0–42.5	209	39.8 ± 1.3	34.4–42.5
Sex (male)	196 (49.9)			110 (52.6)
Child’s race/ethnicity (white)*	287 (74.0)			124 (61.1)
Birth year*
1993–1994	112 (28.5)			81 (38.8)
1995–1996	172 (43.8)			61 (29.2)
1997–1998	109 (27.7)			67 (32.1)
Grade (≥ 2nd)	340 (86.5)			182 (87.5)
Breastfed > 1 month (yes)*	218 (55.5)			91 (44.0)
Prenatal smoking (yes)*	131 (33.3)			34 (20.2)
Prenatal alcohol (yes)	88 (22.4)			23 (18.6)
Prenatal omega 3 (g/month)	393	28.3 ± 14.7	5.3–106.6	117	29.1 ± 15.8	7.3–97.1
Peak blood lead, age 1–3 (μg/dL)	374	6.8 ± 4.1	1.0–38.0	197	6.9 ± 3.6	1.0–22.8
Maternal characteristics
Maternal age at birth (years)	393	26.9 ± 5.3	18.0–40.7	209	26.4 ± 5.6	18.1–41.3
Maternal race (white)*	322 (81.9)			94 (72.3)
Maternal birth place (USA)	313 (79.6)			103 (81.8)
Marital status at birth (married)*	240 (61.1)			98 (52.1)
Parity (first birth)	246 (62.6)			129 (61.7)
Illicit drug use in year before birth (yes)	54 (13.8)			20 (16.1)
Maternal IQ (KBIT)*	393	98.7 ± 10.1	57–124	206	96.6 ± 10.8	68–126
Maternal depression (BDI-II)	391	8.0 ± 8.4	0–49	209	8.8 ± 9.3	0–46
OB risk score	393	15.0 ± 10.1	0.5–52.5	209	16.0 ± 10.8	0–52.8
Household characteristics
Parental education*
Both < high school	28 (7.1)			29 (14.4)
At least one high school	153 (38.9)			79 (39.1)
At least one > high school	212 (53.9)			94 (46.5)
Household income
< $20,000	134 (34.1)			70 (34.0)
$20,000–$40,000	124 (31.6)			75 (36.4)
> $40,000	135 (34.4)			61 (29.6)
HOME score	382	45.7 ± 5.4	28–57	206	45.3 ± 5.4	30–57
OB risk, obstetrical risk score. ^***a***^Children with complete exposure, outcome, and covariate data who were included in our analysis. ^***b***^Children who were excluded from the analysis because of missing exposure or covariate data. ^***c***^Congeners 153, 118, 138, and 180. ^***d***^Congeners 105, 118, 156, 167, and 189. *Significant difference between means of included and excluded participants, *p *< 0.05.						

Three strategies were used to select covariates to include in models: *a*) age at examination and examiner were selected *a priori* to remove potential administration bias and any residual age effects; *b*) variables were selected for which a partial *F*-test indicated that they predicted any of the three outcomes with α < 0.1 when adjusting for all other potential covariates, thereby improving model fit; and *c*) variables were selected whose presence in the model substantially changed the primary exposure effect estimate. This was defined as being ranked in the top three change-in-effect estimates for more than one of the 12 exposure–outcome association models. Covariates included in the final adjusted models were examiner; child age at examination, sex, birth year, school grade, prenatal smoke and alcohol exposure; household income; parental education; and maternal age at birth, IQ, birth place, and pregnancy omega-3 intake. A statistical significance criteria of *p* < 0.05 was used for all analyses, unless otherwise noted.

Among the 607 children included in the 8-year follow-up, 602 had complete WRAML data, 583 of these had cord serum organochlorine measures, 405 of these had hair Hg measures, and 393 of these had complete covariate data. Children with complete outcome, exposure, and covariate data (*n* = 393) were included in these analyses. To examine exposure effect modification, we fit models which included interaction terms between pairs of exposure measures. For interpretability, the primary exposure (either the organochlorine measure or MeHg) was kept continuous, and the secondary exposure was dichotomized into the higher one-third versus the lower two-thirds of exposure. Interactions were also examined between the primary exposures and the following dichotomized sociodemographic covariates: sex, maternal IQ (dichotomized at the median), prenatal smoking (ever vs. never), parental education (< vs. ≥ high school education), income (< $20,000 vs. ≥ $20,000), and breastfeeding (ever vs. never). Particular focus was given to interaction by sex, given the importance of potential sexual dimorphism in sensitivity to prenatal PCB or MeHg exposures (Castoldi et al. 2008; Cordier et al. 2002; Sagiv et al. 2012a, 2012b).

The study protocol was reviewed and approved by the human subjects committees of the Harvard School of Public Health and Brigham and Women’s Hospital (Boston, Massachusetts) and of Southcoast Hospitals Group (New Bedford, Massachusetts). Written informed consent was obtained from all participating families before study evaluation.

## Results

Table 1 shows comparisons of children with complete outcome data who were included in this analysis or excluded because of missing covariate or exposure data. Outcome and exposure values were similar in the two groups. Children included in our analysis had higher maternal IQ, higher parental education, and higher rates of breastfeeding than those excluded. Both children and mothers who were included were more likely to be white than those who were not included. Although mothers of included children were also more likely to have smoked and consumed alcohol during pregnancy, there were considerable missing data for these covariates among those excluded. Sensitivity analyses showed that unadjusted associations between each exposure and outcome pair in the full data set (those with complete outcome data) were comparable with associations observed in the analysis data set (those with complete outcome, exposure, and covariate data) (data not shown).

Our study sample had lower WRAML scores than the test standardization sample (mean ± SD, 100 ± 15) consistent with sociodemographic disadvantage (Table 1). Girls tended to score higher than boys on the WRAML indices (see Supplemental Material, Table S1). Children in the second grade or higher and those with higher household income and parental education, mothers who did not smoke while pregnant, and mothers with higher IQ also tended to perform better on the WRAML (see Supplemental Material, Table S1). Non-Hispanic white children scored higher than children of other races or ethnicities (data not shown).

As expected, the three organochlorine measures were significantly correlated with each other, with Spearman correlation coefficients ranging from 0.59 to 0.89. Organochlorines were also significantly correlated with MeHg, although Spearman correlation coefficients were lower, ranging from 0.33 to 0.41. The three WRAML indices were also significantly correlated with each other, with Spearman correlation coefficients ranging from 0.41 to 0.47.

Table 2 compares the crude and final adjusted models. Prenatal MeHg exposure was associated with lower scores for all three measures of childhood memory and learning. The magnitude of the negative associations increased with adjustment for covariates, indicating negative confounding of the unadjusted estimates. Although the magnitude of the association was similar for all three WRAML indices, the MeHg associations were statistically significant only for the Visual Memory Index (*p* = 0.01, compared with 0.14 and 0.08 for the Verbal Memory and Learning indices, respectively). No significant adverse associations of ΣPCB_4_, dioxin-like PCBs, or DDE with memory and learning were observed (Table 2). Model diagnostics and scatterplots showed that there were a small number of observations (*n* = 1–3) (Table 2) with particularly high exposure values. Although there was a significant positive association between DDE and the Learning Index score before and after covariate adjustment, the adjusted estimate was essentially null after three children with particularly high exposures were excluded from the analysis (DDE levels of outliers: 9.74, 10.26, and 14.93 ng/g serum vs. DDE levels of non-outliers: mean± SD, 0.5 ± 1.1; range, 0.02–3.00 ng/g serum).

**Table 2:** The change in WRAML score associated with a 1-unit increase in serum organochlorines or hair Hg levels: crude,^*a*^ adjusted,^*b*^ and adjusted without outliers.^*c*^

Outcome and exposure measure	Crude β (95%CI)	Adjusted β (95%CI)	Adjusted β without outliers (95%CI)
Verbal Memory Index
∑PCB_4_ (ng/g)^*d*^	–1.3 (–5.3, 2.7)	–1.9 (–6.1, 2.3)	–1.4 (–7.3, 4.5)
TEQ (pg/g lipid)^*e*^	–0.2 (–0.6, 0.2)	–0.2 (–0.6, 0.3)	–0.1 (–0.8, 0.5)
DDE (ng/g)	0.2 (–1.0, 1.3)	0.1 (–1.1, 1.3)	1.8 (–5.4, 1.8)
MeHg (μg/g)	–1.1 (–3.3, 1.1)	–1.7 (–3.9, 0.6)	–1.9 (–4.3, 0.5)
Visual Memory Index
∑PCB_4_ (ng/g)^*d*^	3.2 (–0.8, 7.1)	0.5 (–3.8, 4.8)	–0.2 (–6.2, 5.8)
TEQ (pg/g lipid)^*e*^	0.3 (–0.2, 0.7)	0.0 (–0.4, 0.5)	0.0 (–0.7, 0.6)
DDE (ng/g)	1.1 (0.0, 2.3)	0.6 (–0.6, 1.8)	0.1 (–3.6, 3.7)
MeHg (μg/g)	–0.9 (–3.1, 1.3)	–2.8 (–5.0, –0.6)*	–3.1 (–5.5, –0.6)*
Learning Index
∑PCB_4_ (ng/g)^*d*^	3.2 (–1.1, 7.4)	–0.8 (–5.5, 3.8)	3.3 (–3.1, 9.7)
TEQ (pg/g lipid)^*e*^	0.2 (–0.3, 0.7)	–0.1 (–0.6, 0.4)	0.4 (–0.3, 1.1)
DDE (ng/g)	1.6 (0.4, 2.9)*	1.4 (0.1, 2.7)*	–0.3 (–4.2, 3.6)
MeHg (μg/g)	–0.3 (–2.7, 2.0)	–2.2 (–4.6, 0.2)	–2.4 (–5.0, 0.3)
^***a***^Adjusted for child’s age at examination and examiner (*n *= 393). ^***b***^Adjusted for examiner, child’s age at examination, sex, birth year, school grade, parental education, maternal age at birth, maternal birth place, household income, prenatal smoke exposure, prenatal alcohol exposure, prenatal omega-3 exposure, and maternal IQ (*n *= 393). ^***c***^Adjusted for same covariates as Adjusted β. For ∑PCB_4_, TEQ, and MeHg, *n *= 392; for DDE, *n *= 390. ^***d***^Congeners 153, 118, 138, and 180. ^***e***^Congeners 105, 118, 156, 167, and 189. *p < 0.05.			

**Figure 1:**
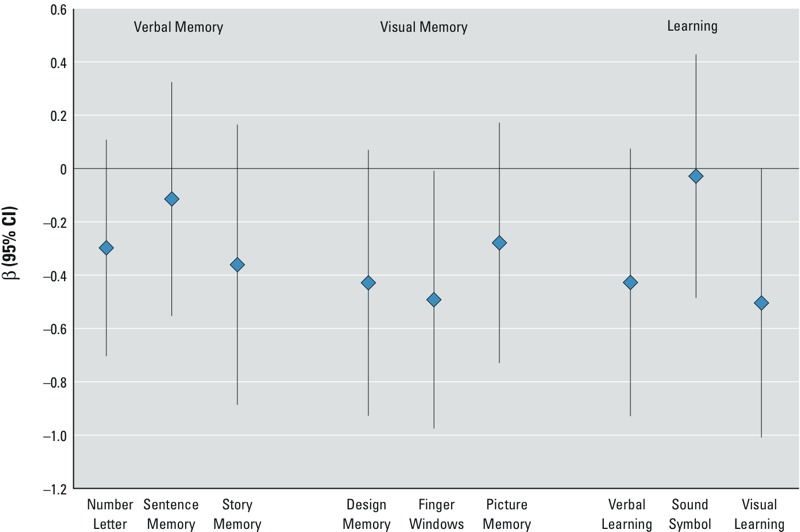
Associations (β and 95% CI) between a 1-μg/g increase in maternal hair Hg and components of the WRAML indices. Estimates from multivariable linear regression models adjusted for examiner, child’s age at examination, sex, birth year, school grade, parental education, maternal age at birth, maternal birth place, household income, prenatal smoke exposure, prenatal alcohol exposure, prenatal omega-3 exposure, and maternal IQ.

Given the associations between MeHg and the WRAML indices, we examined the components of each index to more precisely characterize the exposure–outcome relationships. Adjusted results are shown in Figure 1. Almost all of the subtests showed negative associations with MeHg exposure. In particular, the Finger Windows component of the Visual Index and the Visual Learning component of the Learning Index showed significant negative associations with prenatal MeHg exposure [Finger Windows: β = –0.49, 95% confidence interval (CI): –0.98, –0.01; *p*-value = 0.05; Visual Learning: β = –0.50, 95% CI: –1.01, 0; *p*-value = 0.05].

No significant interactions with the sociodemographic variables (maternal IQ, prenatal smoking, parental education, household income, breastfeeding) were observed (data not shown). Though there were also no significant interactions by sex, consistent with the potential increased sensitivity of boys to MeHg neurotoxicity observed elsewhere (Castoldi et al. 2008; Cordier et al. 2002; Sagiv et al. 2012b), the negative association between MeHg and the Learning Index was stronger in boys than girls (β = –4.1; 95% CI: –7.9, –0.7 for boys vs. β = –0.7; 95% CI: –3.7, 2.4 for girls; *p* for interaction = 0.12). Associations between MeHg and the WRAML indices were comparable when stratified by PCB levels, except for the Verbal Memory Index, wherein suggestive differences by PCB exposure were observed. Specifically, MeHg–Verbal Memory Index associations were negative among children with high (upper tertile) PCB levels (β = –2.5; 95% CI: –5.3, 0.3) and null among those with low (lower two tertiles) PCB levels (β = 0.4; 95% CI: –3.3, 4.2) (*p* for interaction = 0.21). PCB concentrations were not significantly associated with the memory and learning indexes, regardless of whether MeHg levels were high or low (data not shown).

## Discussion

At the exposure levels in our study, after removing outliers, there were no significant associations between prenatal PCB or DDE exposures and memory and learning skills as assessed by the WRAML. Similarly, prenatal PCB exposure was not associated with several learning outcomes that were assessed among school-age children in a Faroe Islands cohort (Grandjean et al. 2001) or in a cohort based in North Carolina (Gladen and Rogan 1991). However, a study conducted on children born to Lake Michigan fish eaters, reported that prenatal PCB exposure was associated with lower scores on the Sternberg, a test of working memory and executive function, at 4 years of age (Jacobson et al. 1992), and the McCarthy memory scale at 4 years of age, which assesses short-term recall (Jacobson et al. 1990).

The PCB exposure levels in our cohort were much lower than those in most other cohorts, including the Michigan cohort (Longnecker et al. 2003). Our null PCB findings may be due to nonlinearity (e.g., a threshold effect) in the dose–response relationship at low exposure levels or perhaps a lack of power. Furthermore, exposure sources may be different in our cohort than in Michigan and other populations selected based on contaminated fish intake (Jacobson et al. 1990; Stewart et al. 1999). The New Bedford population was not selected based on fish consumption. Cord serum PCB levels were related not only to fish consumption, but also to consumption of organ meats, dairy, and demographic and lifestyle factors (Choi et al. 2006). Additionally, the congener profile of PCBs in New Bedford may contribute to differential toxicity because, for example, it is disproportionately high in congener 118 compared with other population-based samples (Korrick et al. 2000).

Hair Hg concentrations were associated with lower WRAML memory and learning index scores, and this negative association was statistically significant for Visual Memory (Table 2). With respect to the components of the Indices, hair Hg was associated with significantly lower scores on the Finger Windows subtest of the Visual Memory Index, in which the child is asked to manually reproduce a demonstrated spatial sequence, and the Visual Learning subtest of the Learning Index, in which the child is asked to recall a fixed number of abstract images on a grid, though associations with some of the other components of each index were nonsignificant but similar in magnitude. This was particularly the case for the Design Memory subtest of the Visual Memory Index and the Verbal Learning subtest of the Learning Index (Figure 1). The Finger Windows, Visual Learning, and Design Memory subtests primarily measure skills related to visual–spatial memory, suggesting that this domain may be particularly sensitive to the effects of low-level prenatal MeHg exposure.

These results are consistent with a study conducted in Hong Kong in which low-dose prenatal MeHg exposure was adversely associated with a visual sequencing task among 6- to 10-year-olds (Lam et al. 2013). Additionally, decreased performance on the Stanford–Binet copying tests, which measures visuospatial performance, was associated with maternal hair mercury in 5- to 6-year-olds in French Guiana (Cordier et al. 2002), and with child hair mercury in a population of 7- to 12-year-olds in the Amazon Basin (Grandjean et al. 1999b). In these latter two cross-sectional studies, children experienced substantial MeHg exposure (geometric mean hair Hg levels ranging from 1.4 to 11.0 μg/g) from consumption of fish contaminated with Hg pollution from gold mining. Furthermore, functional magnetic resonance imaging of Faroese boys (a subsample of a population with mean maternal hair Hg of 4.3 μg/g) showed that for tasks requiring visual processing, adolescents with higher prenatal exposure to MeHg had increased and more widespread brain activation than those with lower MeHg exposures, particularly in the primary visual cortex, suggesting toxicant-related damage to visual association areas (White et al. 2011). Likewise, a study of school-age Inuit from Northern Quebec showed that prenatal mercury (mean cord blood Hg, 4.6 μg/dL) was associated with a reduction in the amplitude of specific visual evoked potentials, suggesting subclinical deficits in visual processing (Ethier et al. 2012). Indeed, the above findings, in combination with other evidence from the literature, suggest that centrally mediated visual functions may be important targets of MeHg toxicity (Burbacher et al. 2005; Dasari and Yuan 2009; Saint-Amour et al. 2006).

In contrast to the associations with visual memory found in our study and others (Cordier et al. 2002; Grandjean et al. 1999b; Lam et al. 2013), no significant MeHg associations were found with the Design Memory subtest of the WRAML Visual Memory Index (Myers et al. 2003) in the Seychelles Islands (mean maternal hair Hg, 6.9 μg/g). However, potential confounding by the beneficial effects of polyunsaturated fatty acids were not taken into account in these Seychelles analyses; this may partly account for null associations (Davidson et al. 2008; Oken et al. 2005).

Associations between hair Hg and outcomes were similar between strata defined by a range of sociodemographic factors. Although the interaction was not significant, MeHg exposure was associated with lower verbal learning scores in children with high PCB exposure, but not among children with low PCB exposure. Enhanced MeHg neurotoxicity in the setting of PCB exposure has been observed elsewhere. For example, in another study population with prenatal PCB and MeHg exposures comparable to New Bedford’s, MeHg was negatively associated with cognitive function at 3 years of age only among children with higher PCB exposure (Longnecker et al. 2003; Stewart et al. 2003).

The New Bedford cohort has been exposed to both PCBs and MeHg, allowing us to investigate potential interactions between these contaminants while accounting for potential nutritional confounders. Biomarkers were available for exposure measurement, and, although we lacked individual cord serum lipid levels for our organochlorine analyses, the lipid content of cord serum is low and relatively constant (Denkins et al. 2000), so this is unlikely to have materially impacted our findings. Additionally, hair may be a less sensitive measure of MeHg than blood (Budtz-Jørgensen et al. 2004), which likely biased associations towards the null.

The New Bedford cohort includes a diverse population with a large representation of lower-income families. Such populations are less studied and perhaps disproportionately exposed to environmental hazards. Results of this study support the finding that prenatal MeHg exposure may be associated with decrements in memory and learning, particularly visual memory. This study contributes to the limited body of literature on the effect of prenatal toxicants on memory and learning, and suggests that further population-based research should be undertaken to better understand the effects of low-level prenatal MeHg exposures on child memory and learning in general, and particularly aspects of memory and learning related to visual processing.

## Supplemental Material

(138 KB) PDF

## References

[r1] Amin-Zaki L, Elhassani S, Majeed MA, Clarkson TW, Doherty RA, Greenwood M (1974). Intra-uterine methylmercury poisoning in Iraq.. Pediatrics.

[r2] Beck A, Steer R, Brown G. (1996). Beck Depression Inventory-II..

[r3] Budtz-Jørgensen E, Grandjean P, Jorgensen PJ, Weihe P, Keiding N (2004). Association between mercury concentrations in blood and hair in methylmercury-exposed subjects at different ages.. Environ Res.

[r4] Burbacher TM, Grant KS, Mayfield DB, Gilbert SG, Rice DC (2005). Prenatal methylmercury exposure affects spatial vision in adult monkeys.. Toxicol Appl Pharmacol.

[r5] Caldwell BM, Bradley RH. (1985). Home Observation for Measurement of the Enviroment..

[r6] Castoldi AF, Onishchenko N, Johansson C, Coccini T, Roda E, Vahter M (2008). Neurodevelopmental toxicity of methylmercury: laboratory animal data and their contribution to human risk assessment.. Regul Toxicol Pharmacol.

[r7] Choi AL Levy JI Dockery DW Ryan LM Tolbert PE Altshul LM 2006 Does living near a Superfund site contribute to higher polychlorinated biphenyl (PCB) exposure? Environ Health Perspect 114 1092 1098; 10.1289/ehp.8827 16835064 PMC1513320

[r8] Cordier S, Garel M, Mandereau L, Morcel H, Doineau P, Gosme-Seguret S (2002). Neurodevelopmental investigations among methylmercury-exposed children in French Guiana.. Environ Res.

[r9] Darvill T, Lonky E, Reihman J, Stewart P, Pagano J (2000). Prenatal exposure to PCBs and infant performance on the Fagan Test of Infant Intelligence.. Neurotoxicology.

[r10] Dasari S, Yuan Y (2009). Low level postnatal methylmercury exposure *in vivo* alters developmental forms of short-term synaptic plasticity in the visual cortex of rat.. Toxicol Appl Pharmacol.

[r11] Davidson PW Kost J Myers GJ Cox C Clarkson TW Shamlaye CF 2001 Methylmercury and neurodevelopment: reanalysis of the Seychelles Child Development Study outcomes at 66 months of age [Letter] JAMA 285 10 1291 1293 11255383 10.1001/jama.285.10.1291-a

[r12] Davidson PW, Myers GJ, Cox C, Wilding GE, Shamlaye CF, Huang LS (2006). Methylmercury and neurodevelopment: longitudinal analysis of the Seychelles child development cohort.. Neurotoxicol Teratol.

[r13] Davidson PW, Strain JJ, Myers GJ, Thurston SW, Bonham MP, Shamlaye CF (2008). Neurodevelopmental effects of maternal nutritional status and exposure to methylmercury from eating fish during pregnancy.. Neurotoxicology.

[r14] Denkins YM, Woods J, Whitty JE, Hannigan JH, Martier SS, Sokol RJ (2000). Effects of gestational alcohol exposure on the fatty acid composition of umbilical cord serum in humans.. Am J Clin Nutr.

[r15] Eskenazi B, Marks AR, Bradman A, Fenster L, Johnson C, Barr DB (2006). In utero exposure to dichlorodiphenyltrichloroethane (DDT) and dichlorodiphenyldichloroethylene (DDE) and neurodevelopment among young Mexican American children.. Pediatrics.

[r16] Ethier AA, Muckle G, Bastien C, Dewailly É, Ayotte P, Arfken C (2012). Effects of environmental contaminant exposure on visual brain development: a prospective electrophysiological study in school-aged children.. Neurotoxicology.

[r17] Gladen BC, Rogan WJ (1991). Effects of perinatal polychlorinated biphenyls and dichlorodiphenyl dichloroethene on later development.. J Pediatr.

[r18] Grandjean P, Budtz-Jørgensen E, White RF, Jorgensen PJ, Weihe P, Debes F (1999a). Methylmercury exposure biomarkers as indicators of neurotoxicity in children aged 7 years.. Am J Epidemiol.

[r19] Grandjean P, Weihe P, Burse VW, Needham LL, Storr-Hansen E, Heinzow B (2001). Neurobehavioral deficits associated with PCB in 7-year-old children prenatally exposed to seafood neurotoxicants.. Neurotoxicol Teratol.

[r20] Grandjean P, Weihe P, White RF, Debes F, Araki S, Yokoyama K (1997). Cognitive deficit in 7-year-old children with prenatal exposure to methylmercury.. Neurotoxicol Teratol.

[r21] Grandjean P, White RF, Nielsen A, Cleary D, de Oliveira Santos EC (1999b). Methylmercury neurotoxicity in Amazonian children downstream from gold mining.. Environ Health Perspect.

[r22] Harada M (1978). Congenital Minamata disease: intrauterine methylmercury poisoning.. Teratology.

[r23] Herrick RF McClean MD Meeker JD Baxter LK Weymouth GA 2004 An unrecognized source of PCB contamination in schools and other buildings. Environ Health Perspect 112 1051 1053; 10.1289/ehp.6912 15238275 PMC1247375

[r24] Hobel CJ, Hyvarinen MA, Okada DM, Oh W (1973). Prenatal and intrapartum high-risk screening. I. Prediction of the high-risk neonate.. Am J Obstet Gynecol.

[r25] Jacobson JL, Jacobson SW (1996). Intellectual impairment in children exposed to polychlorinated biphenyls in utero.. N Engl J Med.

[r26] Jacobson JL, Jacobson SW, Humphrey HE (1990). Effects of in utero exposure to polychlorinated biphenyls and related contaminants on cognitive functioning in young children.. J Pediatr.

[r27] Jacobson JL Jacobson SW Padgett RW Brumitt GA Billings RL 1992 Effects of prenatal PCB exposure on cognitive processing efficiency and sustained attention. Dev Psychol 28 2 297.

[r28] Kaufman A, Kaufman N. (1990). Kaufman Brief Intelligence Test..

[r29] Kershaw TG, Clarkson TW, Dhahir PH (1980). The relationship between blood levels and dose of methylmercury in man.. Arch Environ Health.

[r30] Kim R, Aro A, Rotnitzky A, Amarasiriwardena C, Hu H (1995). K X-ray fluorescence measurements of bone lead concentration: the analysis of low-level data.. Phys Med Biol.

[r31] Korrick SA, Altshul L (1998). High breast milk levels of polychlorinated biphenyls (PCBs) among four women living adjacent to a PCB-contaminated waste site.. Environ Health Perspect.

[r32] Korrick SA, Altshul LM, Tolbert PE, Burse VW, Needham LL, Monson RR (2000). Measurement of PCBs, DDE, and hexachlorobenzene in cord blood from infants born in towns adjacent to a PCB-contaminated waste site.. J Expo Anal Environ Epidemiol.

[r33] Lai TJ, Guo YL, Guo NW, Hsu CC (2001). Effect of prenatal exposure to polychlorinated biphenyls on cognitive development in children: a longitudinal study in Taiwan.. Br J Psychiatry Suppl.

[r34] Lam HS, Kwok KM, Chan PH, So HK, Li AM, Ng PC (2013). Long term neurocognitive impact of low dose prenatal methylmercury exposure in Hong Kong.. Environ Int.

[r35] Longnecker MP Wolff MS Gladen BC Brock JW Grandjean P Jacobson JL 2003 Comparison of polychlorinated biphenyl levels across studies of human neurodevelopment. Environ Health Perspect 111 65 70; 10.1289/ehp.5463 12515680 PMC1241307

[r36] Myers GJ, Davidson PW (1998). Prenatal methylmercury exposure and children: neurologic, developmental, and behavioral research.. Environ Health Perspect.

[r37] Myers GJ, Davidson PW, Cox C, Shamlaye CF, Palumbo D, Cernichiari E (2003). Prenatal methylmercury exposure from ocean fish consumption in the Seychelles child development study.. Lancet.

[r38] Ohba T, Kurokawa N, Nakai K, Shimada M, Suzuki K, Sugawara N (2008). Permanent waving does not change mercury concentration in the proximal segment of hair close to scalp.. Tohoku J Exp Med.

[r39] Oken E, Radesky JS, Wright RO, Bellinger DC, Amarasiriwardena CJ, Kleinman KP (2008). Maternal fish intake during pregnancy, blood mercury levels, and child cognition at age 3 years in a US cohort.. Am J Epidemiol.

[r40] Oken E Wright RO Kleinman KP Bellinger D Amarasiriwardena CJ Hu H 2005 Maternal fish consumption, hair mercury, and infant cognition in a U.S. cohort. Environ Health Perspect 113 1376 1380; 10.1289/ehp.8041 16203250 PMC1281283

[r41] Palumbo DR, Cox C, Davidson PW, Myers GJ, Choi A, Shamlaye C (2000). Association between prenatal exposure to methylmercury and cognitive functioning in Seychellois children: a reanalysis of the McCarthy Scales of Children’s Ability from the main cohort study.. Environ Res.

[r42] Sagiv SK Thurston SW Bellinger DC Altshul LM Korrick SA 2012a Neuropsychological measures of attention and impulse control among 8-year-old children exposed prenatally to organochlorines. Environ Health Perspect 120 904 909; 10.1289/ehp.1104372 22357172 PMC3385436

[r43] Sagiv SK, Thurston SW, Bellinger DC, Amarasiriwardena C, Korrick SA (2012b). Prenatal exposure to mercury and fish consumption during pregnancy and attention-deficit/hyperactivity disorder-related behavior in children.. Arch Pediatr Adolesc Med.

[r44] Sagiv SK, Thurston SW, Bellinger DC, Tolbert PE, Altshul LM, Korrick SA (2010). Prenatal organochlorine exposure and behaviors associated with attention deficit hyperactivity disorder in school-aged children.. Am J Epidemiol.

[r45] Saint-Amour D, Roy MS, Bastien C, Ayotte P, Dewailly E, Després C (2006). Alterations of visual evoked potentials in preschool Inuit children exposed to methylmercury and polychlorinated biphenyls from a marine diet.. Neurotoxicology.

[r46] Salvini S, Hunter DJ, Sampson L, Stampfer MJ, Colditz GA, Rosner B (1989). Food-based validation of a dietary questionnaire: the effects of week-to-week variation in food consumption.. Int J Epidemiol.

[r47] Sheslow D, Adams W. (1990). WRAML: Wide Range Assessment of Memory and Learning: Administration Manual.

[r48] Sokol RJ, Rosen MG, Stojkov J, Chik L (1977). Clinical application of high-risk scoring on an obstetric service.. Am J Obstet Gynecol.

[r49] Stewart P, Darvill T, Lonky E, Reihman J, Pagano J, Bush B (1999). Assessment of prenatal exposure to PCBs from maternal consumption of Great Lakes fish: an analysis of PCB pattern and concentration.. Environ Res.

[r50] Stewart PW, Reihman J, Lonky EI, Darvill TJ, Pagano J (2003). Cognitive development in preschool children prenatally exposed to PCBs and MeHg.. Neurotoxicol Teratol.

[r51] Suzuki T. (1988). Hair and nails: advantages and pitfalls when used in biological monitoring.

[r52] Torres-Sánchez L Schnaas L Rothenberg SJ Cebrián ME Osorio-Valencia E del Carmen Hernández M 2013 Prenatal *p,p´-*DDE exposure and neurodevelopment among children 3.5–5 years of age. Environ Health Perspect 121 263 268; 10.1289/ehp.1205034 23151722 PMC3569679

[r53] Van den Berg M, Birnbaum LS, Denison M, De Vito M, Farland W, Feeley M (2006). The 2005 World Health Organization reevaluation of human and mammalian toxic equivalency factors for dioxins and dioxin-like compounds.. Toxicol Sci.

[r54] Vreugdenhil HJ, Mulder PG, Emmen HH, Weisglas-Kuperus N (2004). Effects of perinatal exposure to PCBs on neuropsychological functions in the Rotterdam cohort at 9 years of age.. Neuropsychology.

[r55] White RF, Palumbo CL, Yurgelun-Todd DA, Heaton KJ, Weihe P, Debes F (2011). Functional MRI approach to developmental methylmercury and polychlorinated biphenyl neurotoxicity.. Neurotoxicology.

[r56] World Health Organization. (1990). IPCS Environmental Health Criteria 101 Methylmercury.

