# Supplementary material for: Prenatal Organochlorine and Methylmercury Exposure and Memory and Learning in School-Age Children in Communities Near the New Bedford Harbor Superfund Site, Massachusetts
Source: Environ Health Perspect. 2014 Aug 6;122(11):1253–9. doi: 10.1289/ehp.1307804 (PMC4216164; doi:10.1289/ehp.1307804)
Supplement: (138 KB) PDF [file ehp.1307804.s001.508.pdf]

Supplemental Material

Prenatal Organochlorine and Methylmercury Exposure and Memory and Learning in School-Age Children in Communities Near the New Bedford Harbor Superfund Site, Massachusetts

Sara T.C. Orenstein, Sally W. Thurston, David C. Bellinger, Joel D. Schwartz, Chitra J. Amarasiriwardena, Larisa M. Altshul, and Susan A. Korrick

Table S1. Performance on the Wide Range Assessment of Memory and Learning stratified by covariates included in models assessing the relation of prenatal contaminants with memory and learning among New Bedford area children with complete data (n=393).

Covariate	N	Verbal Index Mean (95% CI)	Visual Index Mean (95% CI)	Learning Index Mean (95% CI)
**Age at exam**				
Less than 8 years	203	89.1 (87.4, 90.9)	92.2 (90.5, 93.9)	97.3 (95.5, 99.1)
8 years or greater	190	87.5 (85.5, 89.5)	90.1 (88.2, 92.0)	97.8 (95.6, 100.0)
**Gender**				
Male	196	86.3 (84.5, 88.2)*	89.8 (87.9, 91.7)*	97.2 (95.2, 99.2)
Female	197	90.3 (88.5, 92.2)*	92.6 (90.8, 94.3)*	97.9 (96.0, 99.8)
**Birth year**				
1993-1994	112	89.7 (87.1, 92.3)	94.7 (92.2, 97.2)*	101.0 (98.3, 103.6)*
1994-1995	172	87.1 (85.2, 89.1)	89.8 (87.8, 91.7)*	96.4 (94.2, 98.5)*
1995-1996	109	88.8 (86.4, 91.7)	89.8 (87.6, 92.1)*	95.8 (93.4, 98.2)*
**School grade**				
Less than 2nd grade	53	82.9 (79.2, 86.5)*	86.6 (83.1, 90.0)*	91.6 (87.7, 95.5)*
2nd grade or higher	340	89.2 (87.8, 90.5)*	91.9 (90.5, 93.3)*	98.5 (97.0, 99.9)*
**Parental education**				
Both less than HS	28	79.4 (74.0, 84.8)*	85.7 (80.0, 91.3)*	97.3 (91.4, 103.1)
At least one with HS	153	87.3 (85.3, 89.3)*	89.3 (87.2, 91.4)*	97.2 (94.9, 99.4)
At least one greater than HS	212	90.3 (88.5, 92.1)*	93.3 (91.6, 95.0)*	97.8 (96.0, 99.7)
**Maternal age at birth**				
Less than 35 years	368	88.3 (86.9, 89.7)	91.1 (89.7, 92.4)	97.6 (96.1, 99.0)
Greater than or equal to 35 years	25	89.2 (84.8, 93.6)	92.9 (88.4, 97.4)	97.3 (93.1, 101.5)
**Maternal birthplace**				
Not born in US	80	85.9 (83.0, 88.8)*	90.8 (87.5, 94.1)	98.8 (95.4, 102.2)
Born in US	313	89.0 (87.5, 90.4)*	91.3 (89.9, 92.7)	97.2 (95.7, 98.7)
**Household income**				
Less than 20K	134	85.1 (82.9, 87.3)*	89.1 (86.9, 91.4)*	93.9 (91.6, 96.3)*
20K to 40K	124	89.0 (86.7, 91.3)*	90.7 (88.3, 93.0)*	98.7 (96.1, 101.2)*
Greater than 40K	135	91.0 (88.7, 93.2)*	93.7 (91.6, 95.8)*	100.1 (97.9, 102.3)*
**Smoking during pregnancy**				
No prenatal smoke	262	89.2 (87.6, 90.9)	93.0 (91.5, 94.5)*	98.8 (97.1, 100.5)*
Prenatal smoke	131	86.5 (84.3, 88.7)	87.5 (85.1, 89.9)*	95.0 (92.5, 97.5)*
**Alcohol during pregnancy**				
No prenatal alcohol	305	87.9 (86.5, 89.3)	90.3 (88.8, 91.7)*	97.5 (95.9, 99.1)
Prenatal alcohol	88	89.9 (86.8, 93.0)	94.4 (91.5, 97.2)*	97.7 (94.6, 100.8)
**Omega 3**				
Less or equal to than 1 gram/day	240	88.8 (87.2, 90.3)	91.6 (89.9, 93.2)	98.7 (96.9, 100.5)
Greater than 1 gram/day	153	87.7 (85.4, 90.0)	90.6 (88.5, 92.7)	95.7 (93.5, 97.9)
**Maternal IQ (KBIT score)**				
Less than or equal to 80	20	77.3 (71.3, 83.3)*	85.1 (80.4, 89.8)*	94.4 (87.4, 101.3)
Greater than 80	373	88.9 (87.6, 90.3)*	91.5 (90.2, 92.8)*	97.7 (96.3, 99.1)

Abbreviations: KBIT, Kaufman Brief Intelligence Test; HS, high school.

*Significant difference between covariate levels, p<0.05.
